# An antioxidant response is involved in resistance of *Giardia duodenalis* to albendazole

**DOI:** 10.3389/fmicb.2015.00286

**Published:** 2015-04-10

**Authors:** Raúl Argüello-García, Maricela Cruz-Soto, Rolando González-Trejo, Luz María T. Paz-Maldonado, M. Luisa Bazán-Tejeda, Guillermo Mendoza-Hernández, Guadalupe Ortega-Pierres

**Affiliations:** ^1^Departamento de Genética y Biología Molecular, Centro de Investigación y de Estudios Avanzados Instituto Politécnico Nacional, Mexico CityMexico; ^2^Probiomed, TenancingoMexico; ^3^Ingeniería de Biorreactores, Facultad de Ciencias Químicas, Universidad Autónoma de San Luis Potosí, San Luis PotosíMexico; ^4^Departamento de Bioquímica, Facultad de Medicina, Universidad Nacional Autónoma de México, Mexico CityMexico

**Keywords:** *Giardia duodenalis*, albendazole, drug resistance, antioxidant enzymes, sulfhydryl pool

## Abstract

Albendazole (ABZ) is a therapeutic benzimidazole used to treat giardiasis that targets β-tubulin. However, the molecular bases of ABZ resistance in *Giardia duodenalis* are not understood because β-tubulin in ABZ-resistant clones lacks mutations explaining drug resistance. In previous work we compared ABZ-resistant (1.35, 8, and 250 μM) and ABZ-susceptible clones by proteomic analysis and eight proteins involved in energy metabolism, cytoskeleton dynamics, and antioxidant response were found as differentially expressed among the clones. Since ABZ is converted into sulphoxide (ABZ-SO) and sulphone (ABZ-SOO) metabolites we measured the levels of these metabolites, the antioxidant enzymes and free thiols in the susceptible and resistant clones. Production of reactive oxygen species (ROS) and levels of ABZ-SO/ABZ-SOO induced by ABZ were determined by fluorescein diacetate-based fluorescence and liquid chromatography respectively. The mRNA and protein levels of antioxidant enzymes (NADH oxidase, peroxiredoxin 1a, superoxide dismutase and flavodiiron protein) in these clones were determined by RT-PCR and proteomic analysis. The intracellular sulfhydryl (R-SH) pool was quantified using dinitrobenzoic acid. The results showed that ABZ induced ROS accumulation in the ABZ-susceptible *Giardia* cultures but not in the resistant ones whilst the accumulation of ABZ-SO and ABZ-SOO was lower in all ABZ-resistant cultures. Consistent with these findings, all the antioxidant enzymes detected and analyzed were upregulated in ABZ-resistant clones. Likewise the R-SH pool increased concomitantly to the degree of ABZ-resistance. These results indicate an association between accumulation of ABZ metabolites and a pro-oxidant effect of ABZ in *Giardia*-susceptible clones. Furthermore the antioxidant response involving ROS-metabolizing enzymes and intracellular free thiols in ABZ-resistant parasites suggest that this response may contribute to overcome the pro-oxidant cytotoxicity of ABZ.

## Introduction

*Giardia duodenalis* (syn. *G. lamblia, G. intestinalis*) is the protozoan causing giardiasis that is a leading cause of parasitic diarrheal disease in humans and animals. According to estimations of the World Health Organization (WHO), giardiasis accounts for almost a billion cases worldwide with ∼3 billion people living in areas in which the incidence of the infection is around 30% (Escobedo et al., 2010). The disease incidence may increase due to increasing migrations from and to highly endemic countries (Ekdahl and Andersson, 2005) and eventual climate changes (Parkison et al., 2014). The socioeconomic and clinical impact of giardiasis has prompted its inclusion within the WHO’s Neglected Diseases Initiative (Savioli et al., 2006). The control of this infection requires both the inactivation of the infectious cysts disseminated in the environment and the elimination of pathogenic trophozoites that attach to small intestinal epithelium. Fatty to watery diarrhea is a hallmark of acute and chronic giardiasis that in children may result into malabsorption, failure to thrive and deficit in weight gain. Adult asymptomatic carriers are frequently observed (Adam, 2001; Escobedo and Cimerman, 2007).

To date, vaccines for giardiasis are only available for animals and the control of *Giardia* infections in humans relies on improvements in sanitation and hygiene (Olson et al., 2000). Currently available antigiardial drugs include 5-nitro derivatives of imidazole (metronidazole, tinidazole), furan (furazolidone) and thiazole (nitazoxanide) as well as alternative drugs such as acridines (quinacrine, chloroquine), aminoglycosides (paromomycin) and benzimidazoles [albendazole (ABZ), mebendazole]. Although 5-nitro derivatives of imidazole have a high overall efficiency according to non-randomized clinical trials (60–100%; Busatti et al., 2009), therapeutic failures occur in 20% of individuals in spite of the compliance of drug dosage and duration (Upcroft, 1998). The reasons for failures of drug treatment vary among patients and these include: reinfections, inadequate doses in drug treatment, immunosuppression, drug sequestration in the gallbladder or pancreatic ducts, and infections caused by drug resistant in *Giardia* (Nash et al., 2001).

Since 1990, ABZ (methyl [5-(propylthio)-1*H*-benzimidazole-2-yl] carbamate, ABZ), was used in combination with mebendazole to treat giardiasis. Although ABZ is as effective as metronidazole against giardiasis, the efficacy of ABZ may vary significantly (25–90%) depending on the duration of the treatment (1–5 days; Watkins and Eckmann, 2014). Failures of drug treatments for giardiasis have been reported when ABZ is used alone (Kollaritsch et al., 1993; Brasseur and Favennec, 1995; Nash et al., 2001) and when ABZ has been used in combination with metronidazole (Abboud et al., 2001; Mørch et al., 2008). Benzimidazoles are given at single dose to deworm children at 6 month-intervals (i.e., a suboptimal regime for giardiasis) in community- or cohort-based programs in endemic regions of Mexico, Bangladesh, and Bolivia This results in poor elimination of *Giardia* trophozoites (Quihui-Cota and Morales-Figueroa, 2012) and an increase in *Giardia* burdens among the patients (Northrop-Clewes et al., 2001; Blackwell et al., 2013). In this context, *in vitro* studies suggest that ABZ-susceptible and ABZ–resistant *G. duodenalis* trophozoites subpopulations may coexist in culture and continuous exposure to sublethal (e.g., IC_50_) ABZ concentrations could render not only variant proportions of these subpopulations (Argüello-García et al., 2004) but variations in gene expression (Argüello-García et al., 2009). When ABZ concentration is continuously increased in cultures the surviving parasites may be derived from the selection of drug-resistant trophozoites or by the adaptation of drug-susceptible ones. Thus ABZ resistance in giardiasis is an issue of growing concern for public health. In order to identify and analyze the mechanism(s) involved in ABZ resistance in* G. duodenalis,* cultures able to grow in the presence of 1.35, 8 and 250 μM ABZ [minimal lethal concentration (MLC) of parent WB strain: 0.32 μM] were obtained by continuous exposure of trophozoite subculture under increasing sub-lethal drug concentrations (Argüello-García et al., 2009). Since specific mutations at hot-spot amino acid positions (50, 134, 165, 167, 198, and 200) in β-tubulin were associated with ABZ-resistant helminthic parasites (Venkatesan, 1998), these positions were initially analyzed in ABZ resistant *Giardia* clones; however, no mutations were found at these sites (Argüello-García et al., 2009). Further proteomic and RT-PCR analyses showed a subset of seven genes that were upregulated in the ABZ-resistant clones at the protein and mRNA levels. Some of these genes encoded proteins that are involved in cytoskeletal dynamics (alpha-2-giardin, ran-binding protein), energy metabolism (phosphoglycerate kinase and ornithine carbamoyltransferase) and antioxidant response (NADH oxidase; Paz-Maldonado et al., 2013). These data are consistent with the notion that ABZ not only affects parasite microtubules but also glucose uptake (Vinaud et al., 2008) and the induction of oxidative stress in the parasites (Docampo, 1990; Cvilink et al., 2009a). This stress is likely to play an important role in the drug-resistant phenotype since ABZ may be oxidized by phase I enzymes into sulphoxide (ABZ-SO) and sulphone (ABZ-SOO) metabolites that in turn could play a role in the parasite susceptibility or resistance to this drug (Cvilink et al., 2008, 2009b).

In this work we analyzed the pro-oxidant activity of ABZ to assess the production of its metabolites, and to determine the presence and levels of antioxidant components in drug-susceptible and –resistant *G. duodenalis* clones in order to unravel the effector mechanisms involved in ABZ-resistance in this parasite.

## Materials and Methods

### Trophozoite Cultures

The ABZ-resistant clones able to grow under 1.35, 8, and 250 μM concentrations of this drug were obtained as previously described (Argüello-García et al., 2009). A control culture of an ABZ-susceptible clone grown in the presence of 0.5% v/v dimethylformamide (DMF: vehicle), were used throughout this study. Trophozoites were axenically sub-cultured in 4.5 ml screw-capped vials, tubes or bottles at 37°C in modified Diamond’s TYI-S-33 medium (ATCC no. 1404; Keister, 1983). Parasites were harvested at late-log phase by chilling culture tubes on ice for 30 min, washed three times in phosphate-buffered saline (PBS) pH 7.2, counted in a haemocytometer and adjusted to the required cell density.

### Determination of ABZ and ABZ-Metabolite Levels

To assess the conversion of this drug into sulphoxide and sulphone metabolites in ABZ-susceptible and –resistant *G. duodenalis* trophozoites, the intracellular concentration of ABZ species was determined by high performance liquid chromatography (HPLC) in cell lysates obtained at different time periods (0–48 h) after exposure of parasites to ABZ. To standardize this technique, synthetic derivatives of ABZ (sulphoxide, sulphone and amino) and of MBZ were previously analyzed by thin layer chromatography and melting point after dissolving in water:methanol. Then these components were separated (loop: 200 μL) using a mixture of acetonitrile:water at different proportions containing 0.5% v/v acetic acid as mobile phase through a Nucleosil^TM^ C18 column (5 μm particle size, 125 × 4.6 mm) coupled to a binary pump (Waters^TM^ 1525) and a detector with photodiode array (Waters^TM^ 996). In all assays, chromatographic running was carried out for 13-min to separate peaks of each ABZ species and MBZ either in cell-free samples or when added at different concentrations to lysates of non-treated trophozoites. All species were recovered from cell lysates by ethyl acetate extraction followed by solubilisation in methanol/water. The corresponding values of the area under curve (AUC) were analyzed to generate the concentration-AUC calibration curves in which the experimental values of AUCs in lysates from ABZ-treated trophozoites were interpolated. This procedure was used to quantify the intracellular ABZ species. In this assay trophozoites lysates were prepared after incubation of 50 × 10^6^ parasites (ABZ-susceptible and –resistant, previously maintained 5 days in ABZ-free medium) in 15 mL culture tubes in TYI-S-33 medium containing 10 μM ABZ at 37°C for different time periods. Cells were harvested as described above and washed three times with PBS containing 0.2% w/v trichloroacetic acid and three times with PBS alone. After counting parasites cell pellets were resuspended in 180 μL deionized sterile water, frozen at -70°C for 5 min and thawed at 37°C for 5 min and this procedure was carried out five times. Lysates were extracted by adding ethyl acetate (700 μL) and incubating the lysates at 37°C for 45 min. Supernatants were recovered by centrifugation at 750 × *g* for 10 min and dried under vacuum (SpeedVac^TM^ centrifuge). For HPLC analysis, all the test samples were resuspended in 50 μL ethanol, stirred and mixed with 150 μL of MBZ 10 μM in water as internal standard. Results were expressed as micromolar concentration of each ABZ species per 1 × 10^6^ trophozoites.

### Production of Reactive Oxygen Species (ROS) by Trophozoites Exposed to ABZ

To monitor the induction of oxidative stress in ABZ-exposed *G. duodenalis* trophozoites, the Image-iT^TM^ Live Green ROS detection kit (Molecular Probes^TM^, USA) was used with some modifications. This assay is based in the non-fluorescent 5-(and-6)-carboxy-2^′^, 7^′^-dichlorodihydrofluorescein diacetate (carboxy-H_2_DCFDA) that penetrates into the live cells, as deacetylated by non-specific intracellular esterases. This compound upon the presence of non-specific ROS generated in the cell (particularly during oxidative stress) is oxidized and it emits bright green fluorescence that is detected by UV light microscopy using standard fluorescein filters. In these assays, 1 × 10^6^ trophozoites were exposed to ABZ for 24 h at 37°C, washed three times with warm PBS, transferred to 1.5 mL microcentrifuge tubes and labeled in suspension by the addition of 300 μL PBS containing 25 μM carboxy-H_2_DCFDA for 45 min at 37°C in the darkness. Then cells were washed again with PBS, transferred to 10-mL capped polystyrene tubes and resuspended in 200 μL of this buffer prior to analysis by fluorescence microscopy (microscope Zeiss Axioskop 40) and flow cytometry (flow cytometer Beckman FACScalibur II).

### Determinantion of the Expression and Levels of Antioxidant Elements in ABZ-Susceptible and –Resistant Trophozoites

#### Proteomic Analyses

Proteomic assays were carried out as previously reported (Paz-Maldonado et al., 2013). In brief, protein extraction was done on 1 × 10^7^ trophozoites that were sonicated in the presence of protease inhibitors (Complete, Roche^TM^, USA); proteins were extracted with acetone-trichloroacetic acid-β-mercaptoethanol and then were precipitated at -20°C overnight. Pellets were washed with ice-cold methanol or acetone, dried and resuspended in IEF buffer containing urea, thiourea, CHAPS, dithiothreitol (DTT), carrier ampholytes pH 3–10 (Amersham Biosciences^TM^, USA) and bromophenol blue. Protein concentrations were quantified using the Lowry assay with bovine serum albumin as a standard. For two-dimensional electrophoresis, 250 μg of protein were loaded on each IPG dry gel strip (13 cm, pH 3–10; Amersham Biosciences^TM^). After isoelectrofocusing, strips were reduced with DTT and alkylated with iodoacetamide. Following slab gel electrophoresis (12.5% acrylamide gels) protein spots were stained with silver nitrate. Three replicate gels were obtained for each clone analyzed. In this study specific protein spots corresponding to antioxidant enzymes namely NADH oxidase (NADHox), peroxiredoxin 1a (PXR1a), flavodiiron protein (FDP) and superoxide reductase (SOR) were analyzed. As a first approach spots corresponding to the expected molecular weight and isoelectric point of these proteins were defined in stained gels. To identify the selected spots, tandem mass spectrometry (MS/MS) was performed using a 3200 Q TRAP^TM^ hybrid spectrometer (Applied Biosystems/MDS Sciex^TM^, Canada) as described before (Paz-Maldonado et al., 2013). Database searching (NCBI-nr) and protein identification were carried out from the MS/MS spectra using the Mascot Software (http://www.matrixscience.com, Matrix Science^TM^, UK). For image analysis, stained gels were scanned with LabScan software on Imagescanner and analyzed using the Image Master v 5.0 system (Amersham Biosciences^TM^, USA) according to manufacturer’s protocols. The following criteria for differential protein expression were used: a % volume n-fold > 1.0 of spot was considered as increase of expression or a % volume n-fold < 1.0 of spot was considered as decrease of expression in ABZ-resistant clones as compared to sensitive ones.

#### mRNA Levels of Antioxidant Enzymes

In order to analyze the relative levels of mRNA expression of each of the aforementioned antioxidant enzymes in ABZ-resistant or -sensitive *G*. *duodenalis* clones, a standardized retrotranscription-PCR protocol (end point RT-PCR) was used. This technique has already provided data consistent with semi-quantitative proteomics results but with intrinsic higher sensitivity (Paz-Maldonado et al., 2013). RNA was extracted and purified from each culture using the TRIzol reagent (Invitrogen^TM^) and resulting material was used for cDNA synthesis with the SuperScript^TM^ III First-Strand Synthesis System (Invitrogen^TM^) following manufacturer’s instructions. To standardize conditions, both RNA and cDNA were quantified by spectrophotometry (Nanodrop model 2000, Thermo Scientific^TM^, USA). In the PCR reactions, 50 ng cDNA from each clone was amplified using oligonucleotide primer pairs (0.4 μM each primer) that were designed for test gene loci (*nadhox*,* pxr1a*, *fdp* and* sor*) and reference gene loci (protein disulfide isomerase-1 [*pdi1*] and ubiquitin [*ubiq*]). Primer sequences and conditions used for primer annealing and amplification are listed in **Table 1**. In general, primer extension was performed at 72°C for 45 s, except for *nadhox* (1 min), with a final extension step at 72°C for 7 min. After electrophoresis in 2.0% agarose gels, the semi-quantification of amplicons was performed by densitometry using the Image J^TM^ v 1.48 software. The ratio of band intensity (test locus/reference locus) for each ABZ-resistant culture was divided by the corresponding test/reference ratio of the DMF clone to calculate the fold decrease/increase level of expression of each tested gene.

**Table 1:** Loci used as test and housekeeping genes in reverse transcriptase-PCR assays.

Locus	Orf in GiardiaDB (GL50803)	Forward and reverse primer sequences (5^′^–3^′^)	Tm (^∘^C)	Product size (bp)	Cycle number
Peroxiredoxin 1a (*pxr1a*)^a^	16076	AGAAGACCATGGATGCCCGTCCCCATCTCTTCTCC ATGGTCTTCTTGAACGTCT’	55	628	25
NADH Oxidase LTC (*nadhox*)^a^	33769	GTGACGGAGAGGTATGACAAGGTAGTGGGGCTGGAA GAAAAA	55	1020	20
Flavodiiron protein LTC (*fdp*)^a^	10358	AGGAATGGCTCTCTCGTATGACGGTGCTTGCGACTTCA	55	582	25
Superoxide reductase (*sor*)^a^	GLCHR01	CTTTGGCATCACTAAGGAGCTAGAGCTCCATGTAGTTCTC	52	175	25
Ubiquitin (*ubiq*)^b^	7110	GAGCTCATGCAGATCTTCGTCAACCTTCTGGATGGAGTAGT	58	190	25
Protein disulfide isomerase-1 (pdi1)^b^	29487	ACTCCTCTGCTCCTTGTGCTCCTTCGCTGCTTTCAC	55	456	25

*^a^Test gene; ^b^Housekeeping gene; Primer annealing temperature (Tm); Lateral transfer candidate (LTC).*

#### Determination of Free Sulfhydryl (R-SH) Pools

The concentration of free thiols in cell lysates from ABZ-resistant and –susceptible clones was determined using an adapted 5,5^′^-dithio-bis(2-nitrobenzoic acid) (DTNB, Elman’s reagent) method. In this, DTNB reacts with free R-SH groups and releases 2-nitro-5-benzoate which displays a yellowish color with absorbance at^-^ = 412 nm (Riddles et al., 1979). Assays were performed in 1 mL-reactions by suspending 1.2 × 10^7^ PBS-washed trophozoites in 900 μL of 45 mM NaH_2_PO_4_ at pH 7 and adding 100 μL of 1.5 mM DTNB (Sigma^TM^, USA). The mixture was sonicated with six pulses at 12 microns (60% amplitude) with 30 s-intervals until suspension was clear. After incubation at 37°C for 10 min, absorbances were recorded at 412 nm in a SmartSpec 3000 spectrophotometer (BioRad^TM^, USA). To obtain a calibration curve, lysates were substituted for cysteine (stock = 15.5 mM) and experimental absorbances were interpolated to express results as the micromolar concentration of thiols per 1.2 × 10^7^ trophozoites.

### Protein Modeling

The tertiary structure of the antioxidant enzymes assessed in the previous experiments was obtained from amino acid sequences reported in GiardiaDB using the I-Tasser online server (Zhang, 2008; Roy et al., 2010). Sequences in FASTA format were input in I-Tasser which performs structural alignments between query sequences and known templates in the protein databank (PDB) library. This platform retrieves specific parameters for constructed models as the TM score (range: 0–1), an index reflecting the accuracy of alignment for two given structures and considering the root-mean-square-deviation (RMSD) score that indicates a measure of the differences between values predicted by retrieved models and the values observed in PDB templates. As recommended, significant structure alignments when TM > 0.5 were considered. The Cscore (range: -5 to 2) is an index that includes the TM and the RMSD scores and allows to rank the degrees of similarity between two given protein structures.

### Statistical Analyses

In all experiments the data obtained in assays using ABZ-resistant and -susceptible clones were quantitatively compared with the least-square method to linearize calibration curves (Microsoft Excel 2010^TM^) and differences in mean + SD values were assessed by the *t*-student test at a significance of *P* < 0.05.

## Results

### ABZ Metabolites are Differentially Accumulated in *G. duodenalis* Clones Susceptible and Resistant to ABZ

**FIGURE 1:**
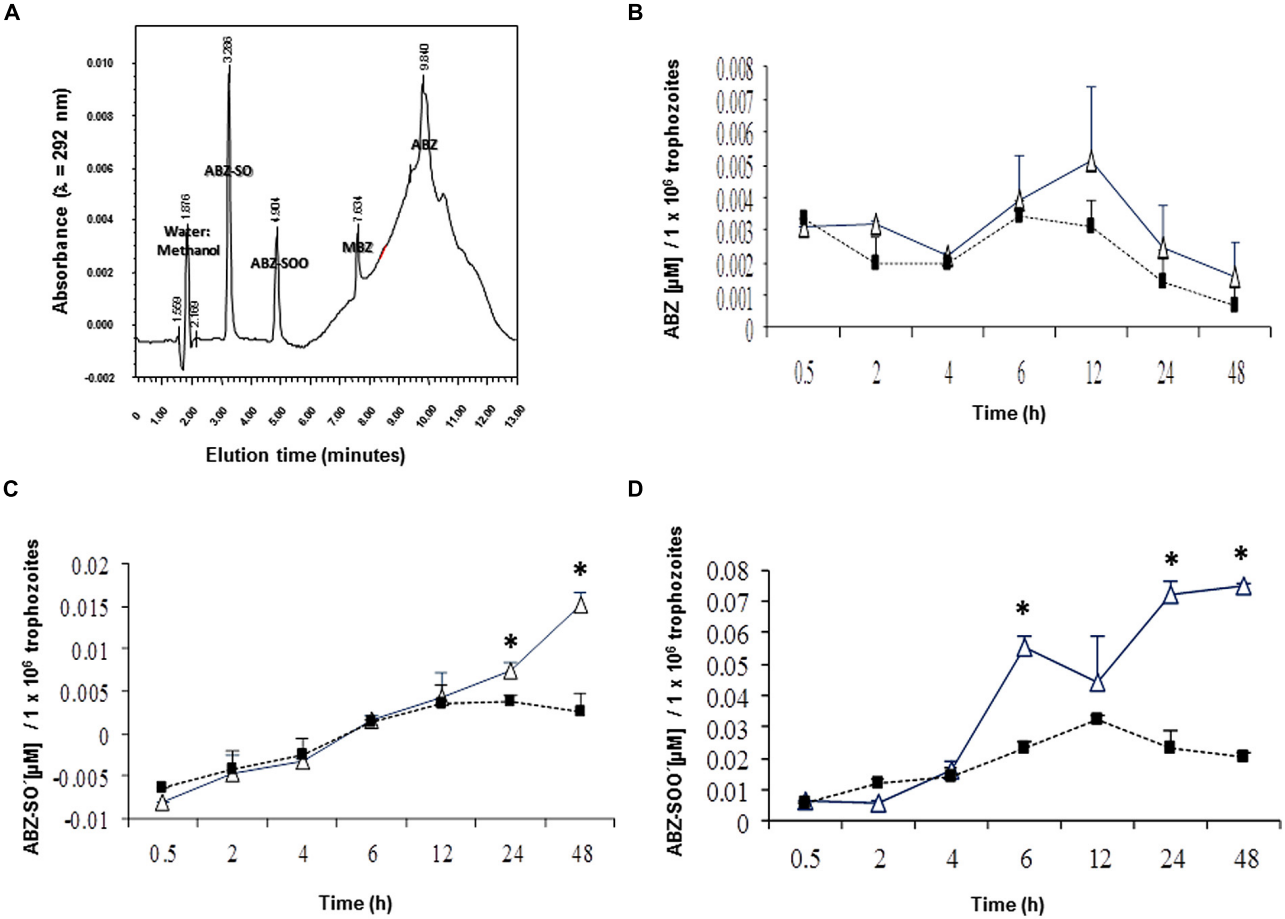
**Differential levels of accumulation of ABZ and ABZ metabolites in *Giardia duodenalis* clones susceptible and resistant to ABZ.** Trophozoites from ABZ-susceptible clone (WBDMF, open triangles) and a representative ABZ-resistant clone (WBR250, filled squares) were exposed to 10 μM ABZ for the times indicated in *X*-axis at 37°C and cell lysates were analyzed by HPLC using MBZ as internal standard. Chromatography assay was standardized using cell-free mixtures, adjusted to 13-min lasting elutions and each ABZ species was separated at the retention time indicated in the corresponding peak **(A)**. These conditions were used in samples of cell lysates and the AUCs were used to determine the levels of ABZ **(B)**, ABZ-SO **(C),** and ABZ-SOO **(D)**. Results are the mean + SD. of three independent experiments and asterisks indicate statistical difference at *P* < 0.05.

In this study, we initially determined if ABZ is metabolized, i.e., oxidized by *G. duodenalis* trophozoites. The ABZ-SO and ABZ-SOO metabolites were identified using a HPLC protocol standardized to detect the three ABZ species and MBZ as the internal standard. A discontinuous gradient, starting with 70% water:30% acetonitrile followed by changes in relative proportions of vehicles to 50:50% (at 5 min), 20:80% (at 8 min), 40:60% (at 10 min) and returning to 70:30% (at 11 min), produced an efficient separation of each ABZ species and MBZ (**Figure 1A**). Under these conditions ABZ species presented maximal emission peaks nearby to 221 and 292 nm (**Table 2**); of these, samples including cell lysates gave low backgrounds at 292 nm hence quantitative chromatograms were obtained at that λ. In addition, the increasing baseline starting at 6-10 min of elution, peaks at 10 min and drops to initial A_292_ values by 12 min (**Figure 1A**) was due to the changing proportions of acetonitrile in the mobile phase and not by artifacts from any of tested compounds, especially ABZ and MBZ for which AUCs were obtained considering these changes in baselines. Likewise controls of ABZ and MBZ-free cell lysates showed this changing baseline with the absence of absorbance peaks and the calibration curves rendered reliable quantifications of each drug species (*R*^2^ ≥ 0.97). Thus, the results showed that ABZ ([ ]_0_ = 10 μM) accumulated at varying concentrations by either drug-susceptible (WBDMF) or –resistant (WBRA250) parasites during the first 12 h of exposure and this concentration dropped by 24–48 h (**Figure 1B**). Although these concentrations were consistently higher in the ABZ-susceptible clone from 2 to 48 h, these differences were not statistically significant, which is likely due to the very short time in which ABZ remains unbound or unmodified. This could also explain the lower levels of ABZ detected in cell lysates as compared to ABZ-SO and ABZ-SOO (see scales in *Y*-axis). On the other hand, the oxidized species ABZ-SO and ABZ-SOO were progressively accumulated by the two clones. However, in the ABZ-resistant clone WBRA250, these species peaked at 12 h and were maintained at similar levels at 24 and 48 h when cells were still viable. At latter times in the WB-DMF clones, an increasing accumulation of ABZ-SO and ABZ-SOO (**Figures 1C,D**) was detected and this was concomitant with generalized cell death.

**Table 2:** Experimental time periods of retention and wavelengths of emission of ABZ species and MBZ (internal standard) as determined by HPLC.

Species	Retention time (min)	λ of emission (nm)	
ABZSO	3.668 ± 0.3	221.1 ± 3.5	288.6 ± 3.2
ABZSOO	5.201 ± 0.3	221.2 ± 2.9	284.5 ± 3.5
MBZ	7.593 ± 0.1	221.1 ± 1.8	305.2 ± 0.4
ABZ	9.459 ± 0.5	222.3 ± 2.4	293.3 ± 1.0

### ABZ Elicits Higher ROS Levels in ABZ-Susceptible Trophozoites

**FIGURE 2:**
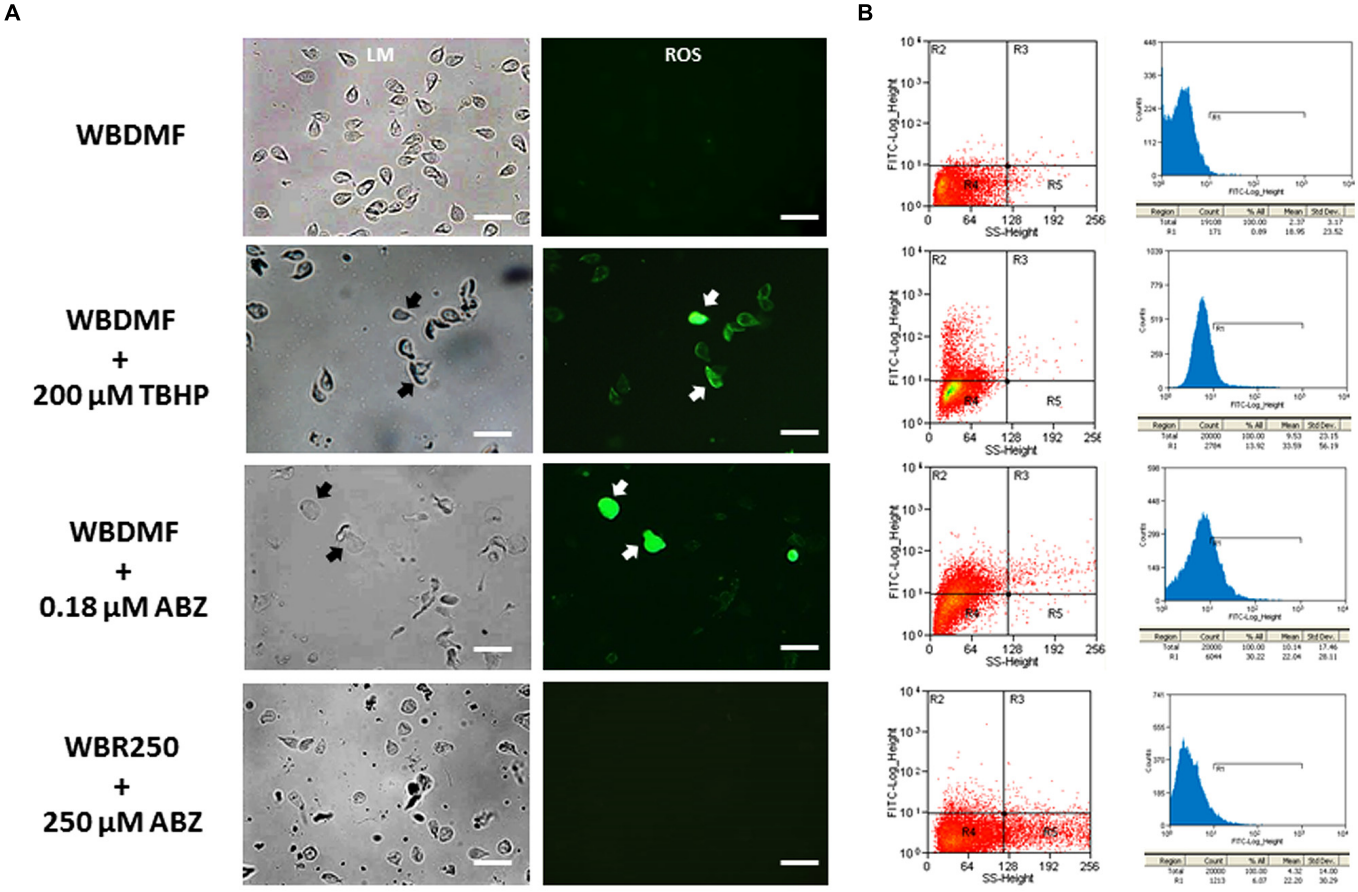
**Determination of ROS produced by ABZ exposure in ABZ-susceptible and ABZ-resistant clones.** Trophozoites from the ABZ-susceptible clone WBDMF or the representative ABZ-resistant clone WBR250 were exposed to ABZ (test) or TBHP (positive control) at the concentrations indicated *at the left* for 24 h at 37°C and observed by light microscopy (LM) and cell fluorescence microscopy (ROS; **A**). The same samples were processed for flow cytometry analysis **(B)** with a window calibrated with fluorescein filters and readings of 20,000 events per sample. Graphs *at the left* are quadrant-based distributions of cells by size/granularity (*X*-axis) and fluorescence intensity (*Y*-axis) and histograms *at the right* show the quantitative displacement of cell fluorescence. Arrows indicate cells displaying profuse ROS staining. Scale bars: 20 μm.

Since ABZ was metabolized into oxidized species, it was of interest to assess if this pro-drug could induce a state of oxidative stress in *G. duodenalis* trophozoites. The use of carboxy-H_2_DCF as a fluorescent ROS tracer allowed the detection of these reactive species by fluorescence microscopy. We observed the distribution of these species in the cytoplasm in the ABZ-susceptible clone (WBDMF), with most cells displaying a punctuate pattern (**Figure 2A**). In the cells with altered morphology (a typical effect of ABZ), bright and diffuse fluorescence was observed (**Figure 2A**, arrows). This latter pattern was also seen in some WBDMF trophozoites exposed to *tert*-butyl hydroperoxide (TBHP), which is another ROS-forming compound (arrows in **Figure 2A**) that was included as positive control because unlike ABZ, TBHP does not alter trophozoite morphology. Interestingly, not all the ABZ-resistant clones displayed significant ROS-associated fluorescence (**Figure 2A**) despite the exposure of these clones to ABZ concentrations (1.35, 8, and 250 μM) that were higher than the one used with the WBDMF clone (0.18 μM). These observations were corroborated by flow cytometry analyses in which the proportions of fluorescent cells were quantified (**Figure 2B**). In quadrant graphics and histograms, we observed the displacement of cell fluorescence (from R4 to R2 and from left to R1 respectively) in the WBDMF clone exposed to ABZ or TBHP, and the absence of such an effect in the representative clone WBR250. In general up to 30% of WBDMF cells displayed ROS fluorescence while in ABZ-resistant clones this proportion was lower (6–9%).

### The Antioxidant Enzymes NADHox and PXR1a are Overexpressed in ABZ-Resistant *G. duodenalis* Trophozoites

**FIGURE 3:**
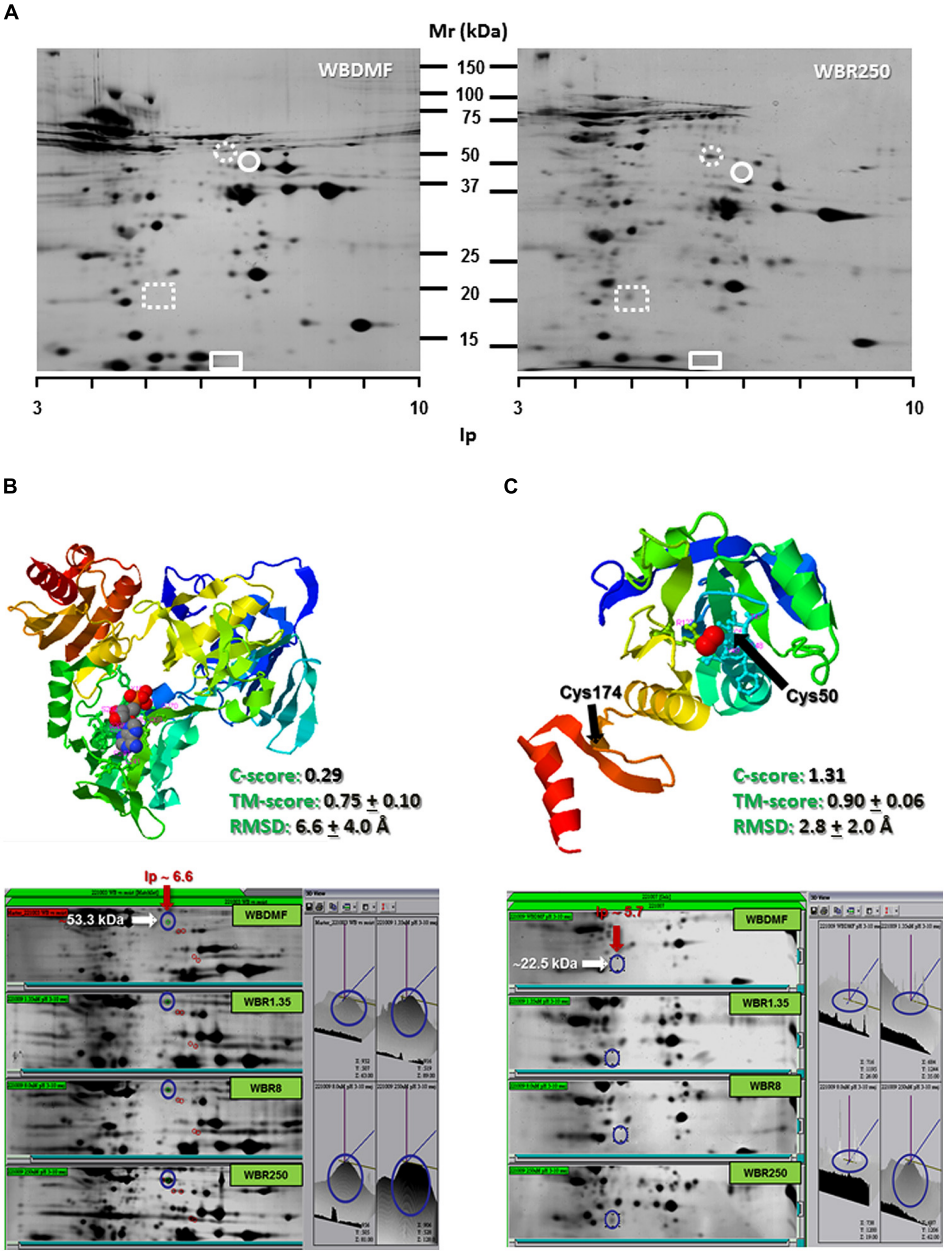
**Determination of NADHox and Pxr1a overexpression in ABZ-resistant *G. duodenalis* clones.** Trophozoite lysates from clones susceptible (WBDMF) and resistant (WBR1.35, WBR8, and WBR250) to ABZ were separated by two dimensional gel electrophoresis **(A)** and the zones matching the expected molecular weight and isoelectric point of NADHox (dashed circle), FDP (circle), Pxr1a (dashed rectangle) and SOR (rectangle) were cut and processed by LC-MS/MS. Proteins detected and identified were NADHox **(B)** and Pxr1a **(C)** and the protein model with the scores of quality parameters is shown in the upper panel. The image analyses of protein spots in representative gels of each clone displaying differential peak volumes are shown in the lower panels. Ligands in protein models (ADP for NADHox and peroxide ion for Pxr1a) are shown in ball conformation.

Since ABZ was able to induce oxidative stress in ABZ-susceptible *G. duodenalis* trophozoites, we examined the expression levels of some antioxidant enzymes in susceptible and resistant parasites. After two dimensional gel electrophoresis, the intensity of protein spots corresponding to antioxidant enzymes as NADHox, PXR1a, FDP and SOR were evaluated by image analysis, and these regions of the gel were also excised for tandem MS/MS analysis. FDP and SOR were elusive to detection because no reproducible spot was found in gel zones nearby to their expected size and isoelectric point (**Figure 3A**). However, NADHox and PXR1a were unequivocally identified as peptides covering 41% of NADHox and 5.7% of Pxr1a amino acid sequences were determined from MS/MS analysis (score > 50; **Table 2**). When the sequences of these two enzymes were submitted to the I-Tasser server, the retrieved protein models are good quality. For NADHox, the model exhibits high degree of structural alignment (75%) and a low structural difference (6.6 Å) with crystallized analogs contained in PDB. For Pxr1a these parameters were even more defined (90% and 2.8 Å respectively; **Figures 3B,C** respectively). In particular, these giardial molecules display maximal structural identity with bacterial and archaeal template homologs: gNADHox with *Lactobacillus sanfranciscensis* NADHox (PDB entry: 2cduB) and gPxr1a with *Aeropyrum pernix* Pxr (PDB entry: 3a2vA). When protein expression levels were determined, the two enzymes detected displayed spots with higher intensity in all ABZ-resistant clones and the WBR250 exhibited the highest increases. Thus NADHox was overexpressed in the fold range of 2.7–3.1 and for Pxr1a this range was of 1.4–3.2 (**Table 3**). These results suggest that at least two antioxidant enzymes are upregulated at the protein level in ABZ-resistant clones.

**Table 3:** Determination of protein expression levels of antioxidant enzymes in *G. duodenalis* clones susceptible and resistant to ABZ.

ID of protein spot	ORF in GiardiaDB (GL50803)	Sequence coverage (%)	MW (kDa)	Ip	R1.35/DMF	R8/DMF	R250/DMF	Expression
NADH oxidase	33769	41	51.0	6.68	3.01	2.71	3.10	Overexpressed
Peroxiredoxin 1a	16076	5.7	21.0	5.34	1.42	1.45	3.29	Overexpressed
Flavodiiron protein	10358	ND	46.6^a^	6.90^a^	ND	ND	ND	ND
Superoxide reductase	GLCHR01	ND	12.5^a^	6.28^a^	ND	ND	ND	ND

*^a^ Theoretical molecular weight (kDa) and isoelectric point (iP).*

### mRNAs of Several Antioxidant Enzymes are Upregulated in ABZ-resistant *G. duodenalis* Trophozoites

**FIGURE 4:**
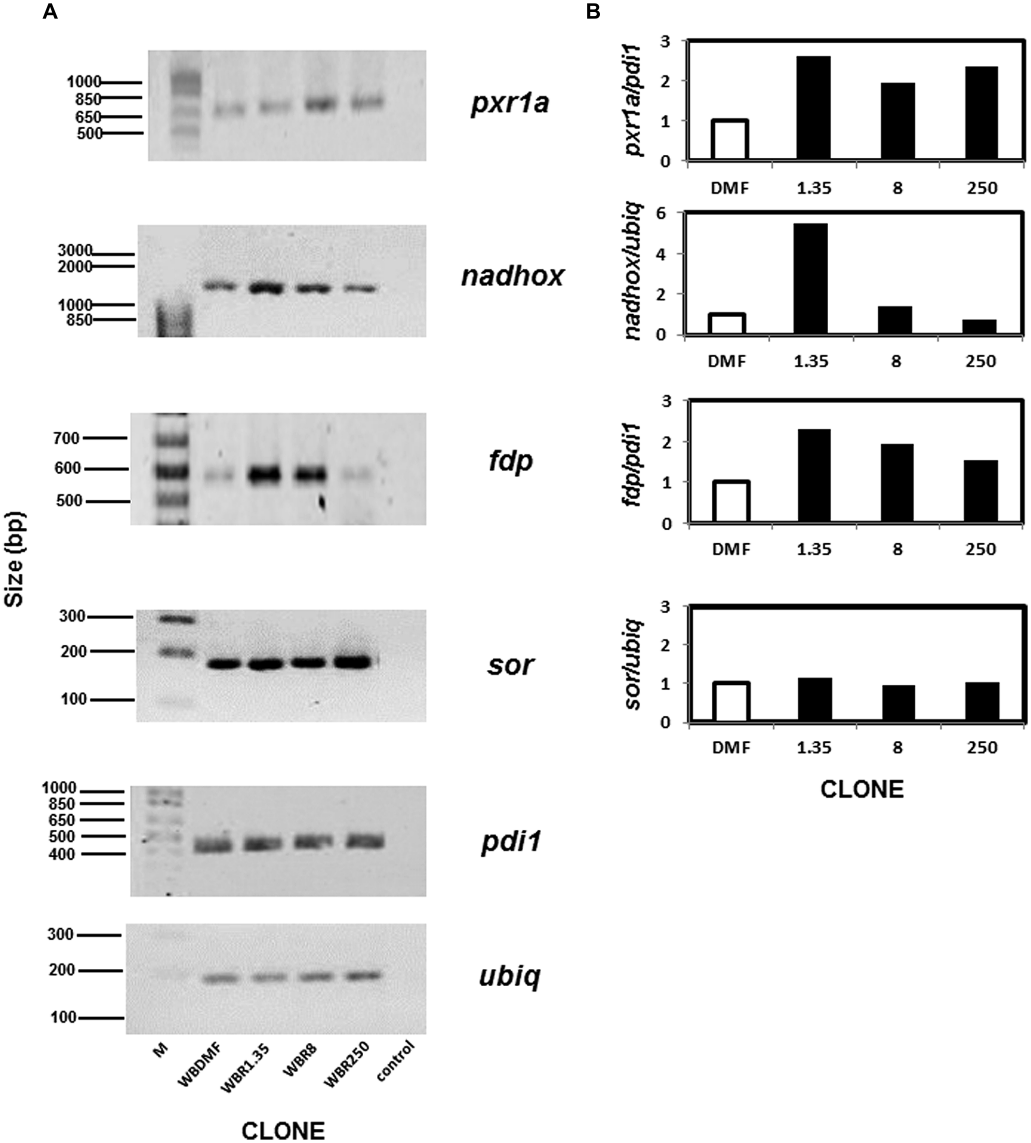
**Determination of mRNAs of antioxidant enzymes analyzed by PCR in ABZ-resistant *G. duodenalis* clones.** Total RNA from trophozoites was reversely transcribed and the resulting cDNA was used to amplify by PCR four test loci that included *pxr1a*, *nadhox*, *fdp* and *sor* and two housekeeping loci (*pdi1* and *ubiq*). Amplicons of expected size were identified by agarose gel electrophoresis in ABZ-susceptible and –resistant clones **(A).** Semi-quantitative densitometry using housekeeping loci to normalize data showed differential patterns of mRNA overexpression in ABZ-resistant clones for all antioxidant enzyme loci except *sor* **(B)**. Data represent the mean values of three independent assays. M, size standards; control, PCR in absence of reverse transcriptase using RNA from the WBDMF clone.

Although we did not observe protein spots on the 2-D gels corresponding to some antioxidant enzymes, this does not necessarily imply that these proteins are absent since they may be present at cellular levels too low for detection by this method. Thus RT-PCR was used to examine the mRNA levels of the genes encoding the antioxidant enzymes and two normalizer genes (*pdi1* and *ubiq*). As expected all the mRNAs from the aforementioned loci could be amplified under the conditions detailed in **Table 1**. The mRNAs for three out of four antioxidant enzymes analyzed were upregulated in ABZ-resistant clones (**Figure 4**). In the case of *pxr1a* mRNA, the clones WBR1.35 and WBR250 displayed increases (>2-fold) that were slightly higher than the one observed in clone WBR8. The increase for *nadhox* mRNA was more pronounced in the clone WBR1.35 (≈6-fold) while the remainder ABZ-resistant clones did not show significant changes. The *fdp* mRNA exhibited an increase as follows: WBR1.35>WBR8>WBR250. In contrast, the *sor* mRNA did not show significant changes among the clones. Among the ABZ-resistant clones displaying mRNA overexpression in the antioxidant enzymes, the WBR1.35 culture exhibited the highest increases of these mRNAs.

### The Antioxidant R-SH Pool Increases in *G. duodenalis* Trophozoites Concomitantly with the Degree of ABZ Resistance

**FIGURE 5:**
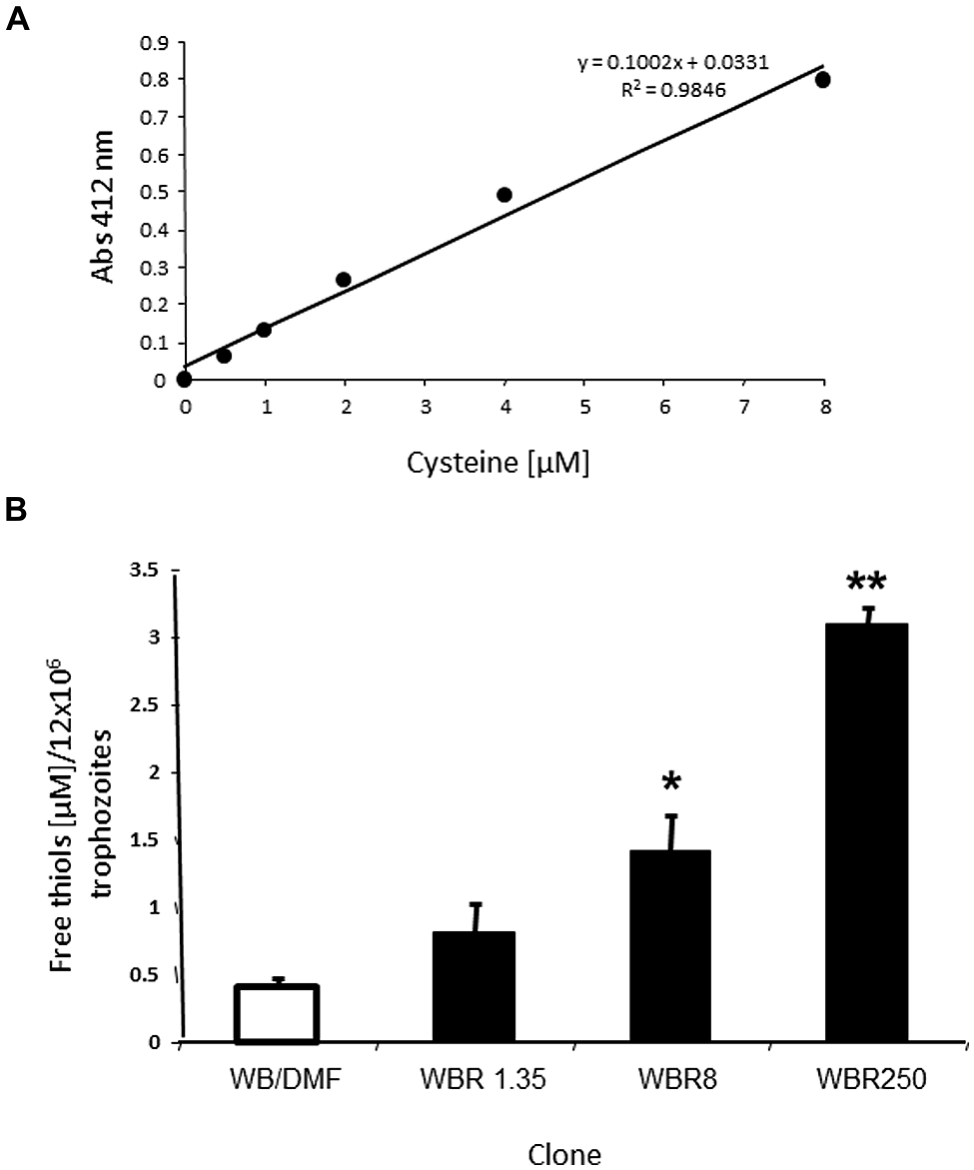
**Differential levels of the sulfhydryl pool in *G. duodenalis* trophozoites susceptible and resistant to ABZ. (A)** Representative calibration curve using cysteine as standard including the corresponding linear regression coefficients. **(B)** Levels of free thiols determined in the ABZ-susceptible clone (open bar) and the ABZ-resistant clones (dark bars) using samples of 1.2 × 10^7^ trophozoites from each trophozoite culture. Data are the mean + SD. of three independent determinations. ^∗^*P* < 0.05, ^∗∗^*P* < 0.01.

Since the antioxidant repertoire of a cell usually comprises enzymes and low MW thiols (R-SH), we were interested in analyzing the R-SH pool in the ABZ resistance model. The thiol groups are antioxidant moieties localized in monomeric (e.g., cysteine) or oligomeric (e.g., glutathione) amino acids or complex polypeptides that can react with the DTNB reagent. We were able to determine the total free thiol pool in trophozoite lysates using calibration curves with reliable accuracy (*R*^2^ > 0.95, **Figure 5A**). Furthermore, it was possible to detect the interaction of DTNB reagent with free intracellular thiols with low backgrounds levels in these assays due to the use of sonication to lyze the cells. In **Figure 5B** are shown the micromolar concentrations of R-SH groups per 1.2 × 10^7^ trophozoites, a cell amount useful to obtain detectable absorbance that could be interpolated in the calibration curves (0.05–0.80). In these assays there were statistically significant increases of R-SH levels in all ABZ-resistant clones as compared to the ABZ-susceptible counterpart. Interestingly, there was an increase in the steady-state R-SH pool levels with increasing ABZ concentrations to which ABZ-resistant cultures were adapted to grow. All together, these data were consistent with the notion that ABZ elicits an antioxidant response in ABZ-resistant trophozoites in which antioxidant enzymes were over- expressed and the increase on the R-SH pools might play an important role in the resistant phenotype.

## Discussion

The pathogenic period of giardiasis involves the establishment of trophozoites into the small intestine milieu of susceptible hosts and the attachment of parasites to the intestinal epithelium. To survive in a hostile microenvironment, trophozoites needs to cope with potentially cytotoxic factors such as dietary microbicides, digestive enzymes, bile salts, fatty acids, mucus as well as the innate or adaptive elements of the host immune response. In this context, *G. duodenalis* is able to display complex responses including antigenic variation, encystation and secretion of metabolic enzymes and proteases (Ankarklev et al., 2010; Lujan, 2011). However, these processes are usually bypassed when effective antigiardial compounds are used (Harhay et al., 2010; Beech et al., 2011; Andrews et al., 2014) since drugs are preferentially directed against parasitic molecule(s) with crucial function in trophozoite survival.

Drug resistance in helminthic and protozoal species such as benzimidazole- or ABZ-resistance has been recognized as a matter of concern in humans and livestock species. To date, the β-tubulin locus in ABZ-resistant *Giardia* generated by *in vitro* subculture is the most extensively model studied (Upcroft et al., 1996; Argüello-García et al., 2009). Since mutations reported in benzimidazole-resistant helminthes (F167Y, E198A, and F200Y) are absent in the giardial β-tubulin locus, it is likely that other mechanism(s) related to drug transport or metabolism are contributing to the resistant phenotype. ABC like eﬄux transporters (e.g., P-glycoprotein, PgP) are unlikely to be involved in ABZ-resistant *Giardia* since this drug, along to MBZ, are not suitable substrates for PgP (Dupuy et al., 2010), and we observed similar kinetics of ABZ accumulation between ABZ-susceptible and -resistant trophozoites in this study (**Figure 1B**). Aside from the direct interaction of ABZ with *G. duodenalis* β-tubulin (MacDonald et al., 2004; Aguayo-Ortiz et al., 2013), these observations suggest an additional cytotoxic role of ABZ as pro-drug against this parasite.

During ABZ metabolism, new cytotoxic species are generated that may induce the development of resistance in *Giardia* trophozoites to this drug. ABZ is a xenobiotic component that in living cells is processed by phase I enzymes (most commonly flavin-containing mono-oxygenases or cytochrome P4540-like,) which insert two oxygen atoms in the sulfur atom at the 5-propylthio group of ABZ in a two-step process (Souhaili-El Amri et al., 1988; Moroni et al., 1995; Cvilink et al., 2009a). Cytochrome P450-like gene families (e.g., *CYP35*) have been identified in helminthes such as *Caenorhabditis elegans* (Menzel et al., 2005), but there are no obvious CYP homologues from searching the GiardiaDB genome database. The resulting sulphoxide (ABZSO) and sulphone (ABZSOO) metabolites still have a parasiticidal effect albeit ABZSO may retain more potency than ABZSOO (Souhaili-El Amri et al., 1988; Bártíková et al., 2010; García-Rodríguez et al., 2012). These oxidized species, particularly ABZSO, have been reportedly detected in several helminthes (Cvilink et al., 2008, 2009a,b; García-Rodríguez et al., 2012; Vokřál et al., 2013). The present study is in agreement with a previous study in which ABZ metabolites were detected in trophozoites by antibody-based microscopy techniques (Oxberry et al., 2000) and confirms that *G. duodenalis* has oxygenases that metabolize both ABZ and ABZSO. Our results that show the greater accumulation of ABZSO and ABZSOO in ABZ-susceptible *Giardia* as compared to its ABZ resistant counterparts (**Figures 1C,D**), is in agreement with recent studies in *Haemonchus contortus* where the S-oxidation of ABZ determined by LC-MS/MS was also lower in the resistant BR strain as compared to the susceptible ISE strain (Vokřál et al., 2013). In this context, it is likely that ABZ-resistant parasites have altered rates of drug metabolization aimed to avoid ABZSO/ABZSOO levels reaching the cytotoxic levels observed in ABZ-susceptible ones.

Albendazole could also be cytotoxic by the generation of oxidative stress in parasites. This phenomenon has been suggested to be induced by other anti-parasitic agents (Docampo, 1990). A hallmark of oxidative stress status is the excessive accumulation of ROS such as oxygen (O_2_), superoxide anion (^.^O_2_^-^), peroxide (^.^O_2_^-2^), hydrogen peroxide (H_2_O_2_), hydroxyl radical (^.^OH), hydroxyl ion (OH^-^). These species are a result of endogenous reactions from mitochondrial [superoxide dismutase (SOD)] or membrane-associated (NADPH oxidase and 5-lipoxygenase) enzymes or from exogenous agents (e.g., pollution, radiation and xenobiotics including drugs). Cellular damage provoked by ROS includes oxidative transformation of DNA, protein aminoacids and lipid peroxidation associated to apoptotic or necrotic cell death. The precise pathway of ROS production by ABZ in mammals and parasites is yet unknown, hence a direct participation of ABZSO and ABZSOO cannot be ruled out as these are S-oxidized species. Moreover benzimidazoles are also able to induce or modify the oxidative stress status in the host (Locatelli et al., 2004; Dimitrijević et al., 2012). In *G. duodenalis*, a microaerophilic organism, oxidative stress may be induced using H_2_O_2_, drugs (metronidazole) as well as cysteine- and ascorbic acid-depleted media. The damage observed varies according to the inducing agent and include cell cycle blockade, apoptotic-like processes (caspase-independent) and changes in gene expression profiles (Ghosh et al., 2009; Raj et al., 2014). In this study, ABZ induced ROS accumulation in susceptible trophozoites as did hydroperoxide, but ABZ had a more marked effect in cell morphology (**Figure 2A**). Therefore, the evidence of oxidative damage to DNA, lipids and proteins, as well as the cell death process elicited by ABZ have been addressed by our group (publication in process). Moreover, the production of ABZ metabolites warrants further studies since three of these metabolites have been detected at distinct locations within ABZ-exposed trophozoites (Oxberry et al., 2000). In contrast, oxidative stress was not observed in ABZ-resistant trophozoites (**Figures 2A,B**), which indicates that an antioxidant response that avoids accumulation of both ABZ metabolites and ROS is associated to the resistant phenotype.

Antioxidant responses are complex but well-orchestrated processes that are evolutionarily conserved from bacteria to higher eukaryotes. These usually involve enzyme systems and molecular antioxidants based on the tripeptide glutathione, single amino acids as cysteine or the R-SH pool that are used to scavenge toxic oxidants and radicals including ROS. *G. duodenalis* belongs to one of the earliest lines of eukaryotic descent and lacks the catalase, SOD, glutathione and glutathione-dependent reductases and peroxidases. However, *Giardia* contains alternative antioxidants such as (a) a NADH oxidase (NADHox) and a FDP (A-type flavoprotein), that can detoxify O_2_ from trophozoites to form water (Brown et al., 1996; Di Matteo et al., 2008); (b) a SOR that converts ⋅O_2_^-^ into H_2_O_2_ (Testa et al., 2011); (c) 2-cys peroxiredoxins (Pxr1a and Pxr1b) with ability to detoxify H_2_O_2_ to form oxygen and water (Mastronicola et al., 2014); and (d) cysteine as the major molecular antioxidant. Based on a *reverse proteomics* (effect-to-cause) strategy to search for the expression of the aforementioned enzymes, two of these enzymes, namely NADHox and Pxr1a, could be detected and were found overexpressed in ABZ-resistant clones. Furthermore, the up-regulation of NADHox, Pxr1aand FDP mRNAs in ABZ-resistant clones suggest that FDP could be overexpressed at the protein level since proteomics and RT-PCR assays gave qualitatively similar results (**Figures 3** and **4**; Paz-Maldonado et al., 2013). The functional significance of changes of expression of antioxidant enzymes (at protein and mRNA levels) could reflect the levels of substrates that are processed by these enzymes, or alternatively, by differences in total catalytical activities between ABZ-susceptible and –resistant trophozoites. In the first scenario, the higher expression of NADHox, Pxr1a and FDP is likely related to a requirement to detoxify O_2_ and H_2_O_2_ under *constitutive* conditions in resistant parasites while ⋅O_2_^-^ levels are not increased under ABZ exposure because SOR mRNA levels were virtually unchanged (**Figure 4**). In the second scenario, the likely increased in the total activity of antioxidant enzymes in ABZ-resistant *Giardia* could be a process associated with resistance to ABZ and other drugs in protozoa and helminthes (Cvilink et al., 2009a; James et al., 2009; Vokřál et al., 2013). Collectively, these observations support the notion that ABZ-resistance in *Giardia* is associated with increased (mRNA or protein) levels of antioxidant enzymes that could cope more efficiently with ROS levels generated during ABZ-induced oxidative stress.

The role of the increased R-SH pool in antioxidant responses and drug resistance has been scarcely studied albeit found in mammalian cell lines, fungal and bacterial models (Cantoni et al., 1994; Cánovas et al., 2004; Song et al., 2013). The requirement of *Giardia* for L-cysteine as a major constituent of its R-SH pool is largely known. In ABZ-resistant trophozoites, the increasing thiol pool observed with the increasing degree of ABZ resistance (**Figure 5**) suggest that another mechanism to scavenge oxidant species generated by ABZ metabolization and/or ROS accumulation besides antioxidant enzymes is present. It is also possible that other drug resistance mechanisms are involved in this phenomenon, such as a higher expression or activity of transporters for antioxidant molecules as L-cysteine.

Taken together, the results from this work emphasize the presence of ABZ biotransforming systems in *G. duodenalis* and the association of increased levels of antioxidant elements (enzymes and R-SH pool) with an efficient antioxidant response in ABZ-resistant trophozoites to avoid accumulation of ABZ metabolites and ROS. This antioxidant response is carried out mostly by archaeal or bacterial-like molecules present in this parasite. Nevertheless it is not expected to be ABZ-specific since peroxiredoxins have been shown upregulated in trophozoites transfected with vectors for neomycin or puromycin selection (Su et al., 2007) and NADHox displayed higher activity in furazolidone-resistant cultures (Brown et al., 1998). Future studies on the regulation of the activity of antioxidant enzymes and the R-SH pool will provide additional insights on the molecular basis underlying ABZ resistance in this parasite and also for drug development in giardiasis.

## Conflict of Interest Statement

The authors declare that the research was conducted in the absence of any commercial or financial relationships that could be construed as a potential conflict of interest.
